# Providing a common language for obesity: the European Association for the Study of Obesity obesity taxonomy

**DOI:** 10.1038/s41366-024-01565-9

**Published:** 2024-06-20

**Authors:** Jacqueline Bowman-Busato, Lucas Schreurs, Jason C. G. Halford, Volkan Yumuk, Grace O’Malley, Euan Woodward, Diederik De Cock, Jennifer L. Baker

**Affiliations:** 1 European Association for the Study of Obesity, Teddington, United Kingdom; 2 Biostatistics and Medical Informatics Research Group, Department of Public Health, Faculty of Medicine and Pharmacy, Vrije Universiteit Brussel (VUB), 1090 Brussels, Belgium; 3 Department of Psychology, University of Liverpool, Liverpool, UK; 4 School of Psychology, University of Leeds, Leeds, UK; 5 Division of Endocrinology, Metabolism and Diabetes, Department of Medicine Istanbul University-Cerrahpasa, Cerrahpasa Medical Faculty, Istanbul, Turkey; 6 Obesity Research and Care Group, School of Physiotherapy, RCSI University of Medicine and Health Sciences, Dublin, Ireland; 7 Center for Clinical Research and Prevention, Copenhagen University Hospital—Bispebjerg and Frederiksberg, Frederiksberg, Denmark

**Keywords:** Public health, Health policy

## Abstract

**Background:**

The basis for a high-performing and resilient healthcare system is having a common, precise, and scientifically accurate language used across all stakeholder groups. However, such a common language is lacking for obesity. Therefore, the European Association for the Study of Obesity undertook a taxonomy initiative to provide standardised language for obesity as commonly used from policy to practice for other major policy-prioritised non-communicable diseases (NCDs).

**Methods:**

An online Delphi consensus study was conducted, involving a panel of experts representing stakeholder groups of policymakers, healthcare professionals, people with lived experience, and researchers. Based on the understanding of obesity as an adiposity-based chronic disease, 54 statements demarcated into definition, scope and contextual usage were developed across six themes: Definition of obesity, Causes, onset and progression, Obesity prevention, Screening and early diagnosis, Treatment and management, Obesity consequences.

**Results:**

Of the 194 invited experts, 70 (36%), 63 (33%), and 58 (30%) experts participated in rounds one, two, and three, respectively. Consensus was achieved on 70% of the proposed definitions, scope, and contextual usage after round one, 94% after round two and 100% after round three. The Definition of Obesity theme included distinctions between population-level indicators and individual-level signs of obesity, and how pre-obesity was defined. The Causes, Onset and Progression theme characterised the timing of obesity development. The Obesity Prevention theme explicitly differentiated between health promotion and primary prevention. Both the Screening and Early Diagnosis, and the Treatment and Management themes defined concepts supporting a continuum of care model. The Consequences of Obesity theme encompassed health and socio-economic outcomes.

**Conclusion:**

The taxonomy provides a contemporary evidence-based language about obesity that aligns with language used for policy-prioritised NCDs. The taxonomy is useful for education, advocacy, and communication and can be used by policymakers, healthcare professionals, people living with obesity, researchers, and health system users.

## Introduction

The basis for a high-performing and resilient healthcare system is one where there is a common, precise, understandable, and scientifically accurate language used amongst and across all stakeholder groups [1, 2]. However, such a common language is lacking for obesity. Although the World Health Organization (WHO) defines obesity as an “abnormal or excessive fat accumulation that can impair health,” the debate regarding the status of obesity as a disease is still polarising in modern medicine, policy and society [3]. An important ramification is that health service delivery systems do not have a chronic disease pathway for obesity embedded in them. The worldwide prevalence of obesity has nearly tripled since 1975, and it is estimated that by 2030 over one billion people globally will be living with obesity [4]. Despite the increasing prevalence and international recognition of obesity as a chronic disease, the language used to describe obesity is often inconsistent, inaccurate, and insufficiently person-centred, which can contribute to misunderstanding, stigma, and discrimination [5, 6].

The importance of language has been highlighted in the field of chronic disease management for obesity and diabetes, and it is a major determinant of the patient-provider relationship, as well as clinical outcomes [7]. The language used by healthcare professionals can impact people living with obesity and those who care for them [8, 9]. Studies indicate the use of inaccurate and inappropriate language can lead to physiological and behavioural changes that contribute to poor metabolic health and unfavourable shifts in body composition [6, 9]. There is a critical gap between the scientific evidence supporting obesity as a neurometabolic disease and a conventional understanding of obesity underpinned by a misconception that obesity is due to “lack of individual will power” and as just being someone’s body size [6, 10]. Misconceptions are further amplified by inaccurate sources of information about obesity from the internet, family, friends, media, and mobile applications [11]. Highlighting this, the Awareness, Care, and Treatment In Obesity maNagement–International Observation (ACTION‐IO) study revealed a need to increase understanding of obesity and to improve education concerning its physiological basis and clinical management [12].

Language used by other stakeholders such as policy makers and regulatory authorities can profoundly impact on how obesity is addressed in the healthcare system from primary prevention, through to diagnosis and screening, treatment, and long-term management. Through initial interaction within the extensive network of the European Association for the Study of Obesity (EASO) [13] it became clear that the scientific evidence for obesity as a chronic disease is not widely circulated amongst stakeholder communities. Policies to date have largely focused on the primary prevention of obesity, but the resulting efforts have been generalised health promotion that crosses all dietary-related chronic diseases, namely cancer, cardiovascular disease and diabetes rather than specific obesity targeted actions [14]. However, there is a critical gap in the attention devoted to other essential aspects of addressing obesity, such as screening, early diagnosis, treatment, and long-term management. This observation aligns with the recommendations put forth by NCD frameworks, which underscores the need for a comprehensive approach. Shared language and a common meaning around key words in a discussion is fundamental to effective communication [15], building mutual understanding and implementation of obesity within the NCD framework. Therefore, the objective of this taxonomy project initiated by the EASO is to provide a common obesity language which can be used by all stakeholders from policy to practice and research.

## Methods

### Study design

We conducted an online Delphi consensus study. This method involved seeking the opinions of a group of experts to assess the extent of their agreement and resolve any disagreements on obesity related terms through discussion and level of consensus [16]. The Delphi-method has been used to establish consensus across a range of subject areas, with several in the field of obesity [17, 18].

For each round of the Delphi survey, an instruction video was made to present the content to all participants. Afterwards, the participants received an email link to an online survey (Appendix 5) in which they were asked to rank the definitions based on their level of agreement with them using a visual analogue scale from (0 ‘not at all’ to 10 ‘very important’). A free-text box was added to each statement in the survey, providing the opportunity to elaborate upon or explain responses. In each round, basic participant demographics of the stakeholder group and country of residence were collected. After each round, the content was updated by incorporating the comments. Following this, each participant received an online survey which only included statements where consensus was not reached in the previous round. Revised statements were presented alongside the original statements from the previous round, the group’s response (percentage agreement/disagreement) and suggestions. For each round, there was an interval of 3 weeks to complete the survey. Delphi participants were invited to every round independent of their response(s) to previous rounds. The Delphi process was stopped for each statement when consensus was reached.

### Research team and Delphi participants

A core research team comprised of EASO representatives, provided strategic input and practical management of the Delphi study, and two independent researchers, who conducted the study set-up, the data collection and performed the analyses. The core research team identified members of the sounding board and Delphi participants based on the following criteria: first, ensuring a broad range of different stakeholders from clinicians (general practitioners, surgeons, physicians), dietitians, industry, nurses, patient advocates, payor community, physiotherapists, policymakers, and researchers. Secondly, experts were invited from different geographical locations within Europe to ensure they were represented. Third, a combination of prominence in the field of obesity and the H-index was used to identify experts in research. Based on this, the sounding board included 14 experts who supported the development of the themes and statements and discussed the survey results. For the Delphi, in addition to the above criteria, leaders of national organisations who are part of EASO were included to ensure input from different national contexts and thus were also invited. We liaised with The European Coalition for People living with Obesity (ECPO) in order to include individuals with the lived experience of obesity in our Delphi survey panel. This process resulted in the identification of 194 experts who were eligible to participate in the Delphi panel.

### Content development

**Fig. 1:**
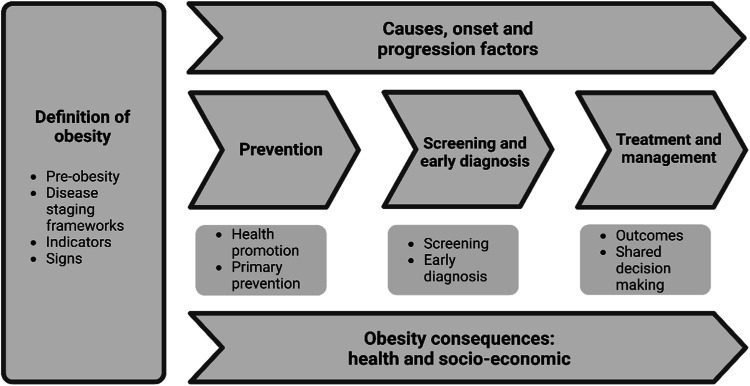
The EASO obesity taxonomy. The EASO obesity taxonomy consists of six themes: Definition of obesity; Causes, onset and progression factors, Obesity prevention, Screening and early diagnosis; Treatment and management; and Obesity consequences. The themes are subdivided into concepts, with each concept further broken down into a definition, scope, and context layer.

Themes and concepts were derived from a literature review centred on the needs and challenges for various stakeholder groups in addressing obesity as a chronic disease [19–21], the “One Voice Exercise: Modified Delphi” among experts on the language and scope of obesity [22], and other NCD frameworks [23, 24]. The core research team developed the structure of the taxonomy based on this evidence and grounded it in the understanding of obesity as an adiposity-based chronic disease. The sounding board was presented with this structure during the first engagement meeting, and they provided feedback through group discussions guided by a decision tree scheme. Based on comments from the sounding board, the taxonomy structure reduced from seven to six themes (Fig. 1) which were: Definition of obesity; Causes, onset and progression factors, Obesity prevention, Screening and early diagnosis; Treatment and management; and Obesity consequences, were formed for the Delphi rounds. Across the themes, 18 concepts were developed, and each concept was further divided into a definition, scope, and context layer, resulting in 54 different statements that had to be ranked.

### Data acquisition and analysis

An online software system (SurveyMonkey) was used to build a secure, web accessible, questionnaire. We instructed the Delphi participants to reflect on their knowledge of obesity and rank each definition on level of agreement by using a visual analogue scale (from 0 ‘not at all’ to 10 ‘very important’). A free-text box was added within each statement of the survey, providing the opportunity to elaborate or explain responses. The six themes were presented in a random order generated by the survey software for the participants. The questionnaire could be completed over several sessions, and there was an option to amend ratings before submitting it. The level of agreement was assessed by quantitative analyses supplemented with a synthesis of the free text responses. The quantitative analysis was performed based on the answers from the scale of agreement (0 not at all to 10 completely agree) and calculated as the percentage of positive responses [7–10] divided by the total completed responses. The free text responses were synthesised by two independent researchers and evaluated for relevance and substance. In the case of disagreement, the core research team resolved this through group discussion.

Responses to the survey were categorised as follows:

- Consensus was defined a priori as ≥75% rates 7–10 and <15% rates 0–3 agreement indicating consensus [25] and no dissenting or relevant comments based on an evaluation of the free text comments.
- Approaching consensus was defined a priori as 60% ≤ x < 75% rates 7–10 OR ≥ 75% rates 7–10 AND > 15% rates 0–3 or dissenting or relevant comments in the free text.
- Half consensus was defined a priori as 50% ≤ x < 60% rates 7 to 10 or dissenting and/or relevant comments in the free text.
- No consensus was defined a priori as <50% rates 7 to 10 or dissenting and/or relevant comments in the free text.

Based on these criteria, the definitions kept being refined for the subsequent rounds of the Delphi study using the following principles:

- If Consensus: include all statements without adjustments.
- If Approaching: include all definitions in the next round, and check if they can be improved by adjusting them based on the rating and comments of statements.
- If Half consensus: include consensus all definitions in the next round, and check if they can be improved by adjusting them based on the rating and comments related to statements.
- If No consensus: create new definitions based on the input from the previous round and/or a new literature search.

## Results

The Delphi survey was conducted between 14 March and 19 September 2023 in three phases. Of the 194 obesity experts who were invited to take part in this three-phase Delphi exercise, seventy (36% participated in round one 63 (33%) in round two and 58 (30%) in round three of the survey. More than 10 professions and 30 countries were represented (based on participants who completed at least one round of the Delphi) (Tables 1 and 2). Out of the 70 involved in round one, 63 and 58 participants participated in rounds, two and three, and 57 (81%), 48 (76%) and 51 (88%) completed the survey in each respective round.

**Table 1:** Demographics of the Delphi panel participants.

Stakeholder group	Round 1 (*n* = 70)	Round 2 (*n* = 63)	Round 3 (*n* = 58)
Clinician	51 (72.9%)	46 (73.0%)	46 (79.31%)
Dietitian	7 (10.0%)	6 (9.52%)	4 (6.90%)
Industry	1 (1.4%)	1 (1.5%)	1 (1.72%)
Nurse			1 (1.72%)
Other^a^	5 (7.1%)	3 (4.8%)	3 (5.17%)
Patient advocate	6 (8.6%)	4 (6.4%)	4 (6.90%)
Payor community			
Physiotherapist	1 (1.4%)		1 (1.72%)
Policymaker		2 (3.2%)	
Researcher	31 (44.3%)	31 (49.2%)	26 (44.83%)

^a^Stakeholders identified themselves using categories. For “other” responses in round 1, participants included a clinical scientist, food policy specialist, metabolic surgeon, psychologist, and a public health specialist. In round 2, this category included an EASO Early career network member, psychologist and a public health specialist. In round 3, this included an academic, food policy specialist, and a medical doctor.

**Table 2:** Delphi panel members by country.

Country	Round 1 (*n* = 70)	Round 2 (*n* = 63)	Round 3 (*n* = 58)
Austria	4 (5.7%)	3 (4.8%)	1 (1.7%)
Azerbaijan		1 (1.6%)	
Belgium	2 (2.9%)	2 (3.2%)	3 (5.1%)
Bosnia	1 (1.4%)		1 (1.7%)
Croatia	1 (1.4%)	1 (1.6%)	
Czech Republic	1 (1.4%)	1 (1.6%)	1 (1.7%)
Denmark	4 (5.7%)	3 (4.8%)	
Finland		2 (3.2%)	
France	1 (1.4%)	3 (4.8%)	1 (1.7%)
Germany	3 (4.3%)	1 (1.6%)	3 (5.1%)
Greece		1 (1.6%)	
Hungary	1 (1.4%)	1 (1.6%)	2 (3.4%)
Iceland			2 (3.4%)
Ireland	3 (4.3%)	2 (3.2%)	5 (8.6%)
Israel	1 (1.4%)	2 (3.2%)	4 (8.9%)
Italy	11 (15.7%)	5 (7.9%)	7 (12%)
Lithuania	1 (1.4%)	2 (3.2%)	1 (1.7%)
Netherlands	1 (1.4%)	2 (3.2%)	3 (5.1%)
Norway	1 (1.4%)	1 (1.6%)	2 (3.4%)
Poland	2 (2.9%)	2 (3.2%)	2 (3.4%)
Portugal	5 (7.1%)	3 (4.8%)	1 (1.7%)
Romania	3 (4.3%)	2 (3.2%)	
Serbia		2 (3.2%)	1 (1.7%)
Slovakia	2 (2.9%)	1 (1.6%)	
Slovenia		1 (1.6%)	
Spain	4 (5.7%)	3 (4.8%)	1 (1.7%)
Sweden	3 (4.3%)	4 (6.3%)	2 (3.4%)
Switzerland	2 (2.9%)	2 (3.2%)	1 (1.7%)
Turkey	5 (7.1%)	2 (3.2%)	7 (12%)
United Kingdom	8 (11.4%)	8 (12.7%)	6 (10.3%)

Table 3 shows the panel’s aggregate rankings for each definition by round. Of the 54 proposed definitions in round one, consensus was reached for 51 (94%) of the definitions, and the remaining three were classified as approaching, half and no consensus per the predefined thresholds. Based on a synthesis of the comments, these three plus an additional 13 definitions did not reach consensus during round one. The absence of consensus was most apparent in Theme 1 (Definition of obesity) and Theme 2 (Causes, Onset and Progression factors) (Appendix Table 1). These comments centred on issues such as “incorrect tone”, “not sufficient”, “too complicated”, “defining the wrong concept”. The 16 definitions were revised by the core team and distributed for round two. The 63 members involved in round two reached consensus on 13 out of 16 definitions per the predefined thresholds, with the remaining three items classified as Approaching consensus. Based on the assessment of the comments, these same three definitions from round two did not obtain consensus (Appendix Table 2). These comments centred on issues such as “too vague”. Again, these definitions were revised by the core team. In round three, 58 members participated in evaluating the remaining 3 definitions and they reached consensus both in the rankings and the comments. Thus, after three rounds of the Delphi process 54 definitions reached consensus.

**Table 3:** Aggregate rankings of the definitions by the Delphi participants for each round.

EASO obesity taxonomy	Round 1		Round 2		Round 3	
**Theme 1: Definition of obesity**	*n*	Rank 7–10	*n*	Rank 7–10	*n*	Rank 7–10
***Concept: Obesity definition***						
• Definition: Obesity is defined as an abnormal or excessive fat accumulation that can impair health.	62	93%				
• Scope: Obesity is an adiposity-based chronic disease which is characterised by the function, total amount and distribution of adipose tissue. Obesity is a disease that consists of different phenotypes.	63	89%				
• Context: the onset, development and progression of obesity can be influenced by a single or many causes or progressing factors.	64	95%				
***Concept: Indicators of obesity***						
• Definition: a metric describing the presence of the disease obesity at the population level.	62	79%				
• Scope: indicators of obesity signal the presence of the disease at the population level.	63	33%	49	84%		
• Context: the following non-exhaustive list of indicators of obesity can be examined: developmental and medical history, laboratory analyses, physical examination at the organ and system levels, mental health, medication intake, activities and tasks of daily living, human behaviours, participation in society, and human exposures.	61	88%				
***Concept: Obesity disease staging frameworks***						
• Definition: Obesity disease staging frameworks classify patients based on signs and indicators, to determine an individual’s health risk and optimise obesity treatment and management.	64	86%	48	83%		
• Scope: staging frameworks are used in many diseases to help to determine disease severity and support clinical decision-making with regards to treatment and management.	64	94%				
• Context: available obesity disease staging frameworks reflect disease frameworks used for other NCD’s.	61	84%				
***Concept: Pre-obesity***						
• Definition: the state of health in which an individual exhibits changes in the function, total amount and/or distribution of adipose tissue that may be a precursor to obesity.	63	76%	49	82%		
• Scope: the state of health in individuals with altered function, total amount and/or distribution of adipose tissue before pathological signs emerge.	64	59%	49	76%		
• Context: overweight does not always reflect the dysregulation of adipose tissue in individuals and therefore is not a synonym for pre-obesity.	65	72%	49	71%	52	81%
***Concept: Signs***						
• Definition: a manifestation of the disease obesity at the individual level	62	79%	49	74%	52	87%
• Scope: physiological, functional or psychological health impairments exist that may signal the presence of obesity in an individual.	63	79%	48	85%		
• Context: at the individual level, an investigation of signs of obesity includes more than just measuring weight or BMI.	63	95%				
**Theme 2: Causes, Onset and Progression factors**	*n*	Rank 7–10	*n*	Rank 7–10	*n*	Rank 7–10
***Concept: Causes***						
• Definition: an event, condition, characteristic or combination thereof which starts the onset of obesity.	64	80%				
• Scope: factors that produce malfunctioning adipose tissue, an abnormal distribution and/or an excessive amount of adipose tissue.	64	82%	52	81%		
• Context: causes of developing obesity may be biologically modifiable or biologically non-modifiable.	64	89%				
***Concept: Onset***						
• Definition: the disease onset is a given moment in time when changes occur that alter the function, distribution and/or total amount of adipose tissue.	64	75%	51	75%		
• Scope: the onset of obesity is the start of the processes that lead to the manifestation of obesity.	62	77%	52	79%		
• Context: the biological processes that provoke the onset of obesity may be ongoing for a long period of time before they are detected.	64	94%				
***Concept: Progression factors***						
• Definition: an event, condition, or characteristic or combination thereof that exacerbates obesity.	64	77%				
• Scope: factors that increase the severity of obesity by altering the biology of the disease.	65	85%	52	73%	51	92%
• Context: factors that cause the progression of obesity may be biologically modifiable or biologically non-modifiable.	62	85%				
**Theme 3: Obesity prevention**	*n*	Rank 7–10	*n*	Rank 7–10	*n*	Rank 7–10
***Concept: Health promotion***						
• Definition: Health promotion is the process of enabling and supporting people and populations to maximise their health and quality of life.	65	97%				
• Scope: Health promotion is generally a behavioural approach to supporting a healthy lifestyle for all.	63	80%				
• Context: Health promotion is delivered to the general public and not only those who might be at risk of obesity.	64	90%				
***Concept: Primary prevention***						
• Definition: Primary prevention aims to prevent the disease of obesity before it ever occurs.	64	92%				
• Scope: Primary prevention targets risk factors of obesity compared to health promotion which enables people to increase, and to improve, their health.	63	80%	50	84%		
• Context: Primary prevention is distinct from secondary prevention, which means early detection, diagnosis and treatment as to stop the progression of obesity and the development of health consequences, and tertiary prevention which means treating and managing the disease of obesity to reduce its long lasting effects.	63	92%				
**Theme 4: Screening and Early diagnosis**	*n*	Rank 7–10	*n*	Rank 7–10	*n*	Rank 7–10
***Concept: Obesity screening***						
• Definition: Screening for obesity refers to the investigation of obesity indicators in populations as to identify individuals with signs of having obesity.	62	87%				
• Scope: elements to consider when screening for indicators for obesity may include a person’s age, biological sex, body composition, ethnic background, family history, pre-existing medical conditions, among others.	61	82%	50	80%		
• Context: Obesity screening can lead to the identification of factors that change the likelihood of developing obesity and use of this knowledge to prevent or lessen obesity by modifying these factors.	63	81%				
***Concept: Obesity early diagnosis***						
• Definition: Early diagnosis of obesity refers to detecting an individual who is living with obesity as early as possible based on signs of this disease.	62	90%				
• Scope: elements to consider when evaluating signs for the early diagnosis of obesity may include a person’s age, biological sex, body composition, ethnic background, family history, pre-existing medical conditions, among others.	57	79%	50	84%		
• Context: Early diagnosis of obesity can lead to better control of disease and to better patient-centred health outcomes, medical outcomes, and socio-economic outcomes in the long term.	61	93%				
**Theme 5: Treatment and Management**	*n*	Rank 7–10	*n*	Rank 7–10	*n*	Rank 7–10
***Concept: Obesity treatment***						
• Definition: healthcare given to a patient living with obesity by a healthcare professional.	65	78%	53	94%		
• Scope: treatment options for obesity or a combination thereof include: Therapeutic physical activity and rehabilitation, Therapeutic nutrition, Psychological therapy, Pharmacotherapy, Metabolic and bariatric surgery	65	94%				
• Context: medical obesity treatment options take a multidisciplinary and holistic approach, and if possible, are person-centred and individualised. Obesity treatment options can be categorised as acute or long-term and consider the fluctuating nature of the disease.	63	92%				
***Concept: Obesity management***						
• Actions taken by individuals, families and communities to promote, maintain and restore health in people living with obesity.	64	88%				
• Scope: Obesity management consists of different levels including: supported self-management, clinical support, informal support and support from the overarching health and social security system.	65	95%				
• Context: Obesity management focusses on rebalancing the biological dysregulation, improving signs and symptoms and thus optimising patient-centred health outcomes, medical outcomes and socio-economic outcomes. Weight management may be a component of this.	63	87%				
***Concept: Obesity treatment and management outcomes***						
• Evaluation undertaken to assess the results or consequences of treating and managing obesity.	63	89%				
• Scope: when treating or managing obesity, patient-centred health outcomes, medical outcomes, and socio-economic outcomes are assessed.	63	94%				
• Context: Obesity treatment and management outcomes go beyond weight.	64	98%				
***Concept: Shared decision-making***						
• Definition: a process in which both the patient and the healthcare professional work together to decide the best plan of obesity care for the patient.	62	98%				
• Scope: the conversation brings together: the clinician’s expertise, such as treatment options, evidence, risks and benefits; what the patient knows best, their preferences, personal circumstances, goals, values and beliefs.	62	98%				
• Context: shared decision-making forms the basis of a clinical consultation. Implementation of this process is useful for complex medical decisions.	63	98%				
**Theme 6: Obesity consequences**	*n*	Rank 7–10	*n*	Rank 7–10	*n*	Rank 7–10
***Concept: Obesity health complications***						
• Definition: Obesity is a gateway disease to a range of medical and mental complications.	62	92%				
• Scope: 200 + complications are associated with obesity including: Type 2 diabetes, heart disease and cancer.	61	94%				
• Context: Obesity was regarded as a comorbidity of many non-communicable diseases whereas now these diseases are seen as medical complications of obesity.	63	89%				
***Concept: Obesity socio-economic consequences***						
• Definition: Obesity can harm an individual’s education, income, job opportunities and value creation.	62	98%				
• Scope: Obesity impacts individuals at several socio-economic levels and decreases their quality adjusted life years.	57	97%				
• Context: Obesity places a significant pressure on public and specialised health institutions in terms of costs and quality of services.	61	83%	50	88%		

Achieving consensus proved most challenging for Theme 1 (Definition of Obesity) and Theme 2 (Causes, Onset, and Progression Factors) (Table 3). Three statements (concept of pre-obesity, context layer; concept of signs, definition layer; concept of progression factors, scope layer) required three rounds before reaching consensus. Six concepts (Obesity Definition, Health Promotion, Obesity Management, Obesity Treatment and Management Outcomes, Shared decision-Making, Obesity Health Complications) only required one round to reach consensus for their definition, scope, and context layers. Thirteen statements, which initially reached consensus through quantitative analyses (≥75% rates 7–10 and <15% rates 0–3) were rephrased and circulated for a second and/or third Delphi round, as they did not achieve consensus, based on the qualitative analyses (Table 3).

As a result of the process, some notable elements emerged. In the Definition of Obesity theme, it emerged that although there is agreement about the definition of obesity, the definition of pre-obesity (e.g. overweight) was more challenging. Even though the terminology aligns with that used for other policy-prioritised NCDs (e.g. diabetes), the shift in language, the subtleties of the shift in focus to adipose tissue rather than weight were not agreed as readily. In the Causes, Onset and Progression Factors theme, the concept of progression factors was most challenging for consensus agreement. Although the scope layer ultimately achieved 92% consensus, there was nuance and ambiguity related to the included factors that increase the severity of obesity. For the Obesity Prevention theme ultimately, a clear distinction was made between health promotion and primary prevention. It took two rounds of the Delphi to make the distinction for the scope layer of primary prevention, and even then, the definition only achieved an 84% consensus. The fourth theme of Screening and Early Diagnosis presented challenges for defining the scope of each of the concepts. Ultimately a compromise was reached between having a fully exhaustive list of elements versus having guiding principles. For the Treatment and Management theme, there was a focus on what treatment actually consists of. A 94% consensus was reached for a definition that used the broad term of “healthcare” delivered by a “healthcare professional”. For the theme of Obesity Consequences, although consensus was always high, there were concerns raised about potential misinterpretations of the wording that obesity places pressure on health care costs, but not the veracity of the statement.

## Discussion

This Delphi survey achieved consensus on 54 statements, which results in a taxonomy that can be used to support and align the way we speak about obesity using the standard vocabulary of policy-prioritised NCDs.

The taxonomy was structured around six main themes which were derived from language frameworks used in other NCDs [23, 24]. Obtaining consensus was most challenging in Theme 1 (Definition of obesity) and Theme 2 (Causes, Onset and Progression factors) (Table 3). These results reflect the ongoing discussion regarding the status of obesity as a chronic disease [7]. The obesity taxonomy has a particular focus on bridging the gap between scientific knowledge and the conventional narrative around obesity, as there is a great need to increase the understanding of obesity and to improve education concerning its physiological basis [12]. The obesity taxonomy harmonises the language of obesity with that of other policy-prioritised NCDs to support the recognition of obesity as an adiposity-based chronic disease which is characterised by the function, total amount, and distribution of adipose tissue [19].

An important element of the taxonomy is that it makes a distinction between the individual level and the population level. For example, within theme 1, the concept of indicators applies to the presence of obesity at the population level whereas the concept of signs applies to the presence of obesity at the individual level. These concepts are useful when interpreting body mass index (BMI; kg/m^2^) which is a useful indicator for screening and epidemiological studies but is less useful at the individual level. Importantly, BMI on its own is insufficient for making a clinical diagnosis of obesity as the limitations of BMI in identifying adiposity at the individual level are well known [10]. These observations are reinforced in our study by a substantial consensus level of 95% regarding the following definition: “At the individual level, an investigation of signs of obesity includes more than just measuring weight or BMI”. To shift the emphasis away from weight and BMI, the terms “function” and “distribution” were included into the definition of pre-obesity and obesity (theme 1). By using the term “pre-obesity,” we harmonise the terminology of obesity with that used by other policy prioritised non-NCDs, such as the term “pre-diabetes” [23, 24, 26]. Further, this term aids in the important distinction that overweight (BMI ≥ 25) focuses on total weight and therefore cannot be used as a synonym for pre-obesity as it does not reflect the dysregulation of adipose tissue.

Within theme 3, obesity prevention, we highlight the critical difference between health promotion and primary prevention. Implementing these concepts into the obesity taxonomy addresses the misconception that health promotion, which is delivered to the general public and not only those who might be at risk of obesity, is the same thing as obesity primary prevention which targets risk factors for obesity to reduce the prevalence of obesity. As such, phrases such as ‘eat less, move more’ and “live a healthy lifestyle“ are often is seen as being primary prevention of obesity, but these are actually health promotion and applicable to all dietary-related chronic diseases. In a recent survey of 5623 respondents across four countries, 79% and 80% of people reported believing that obesity could be prevented and cured by adhering to a healthy lifestyle, reflecting a lack of understanding of obesity as an adiposity-based chronic disease [27]. Although a healthy lifestyle is beneficial for everyone, this ignores the scientific evidence of obesity being a neurometabolic disease that varies in its severity, thus requiring different levels of treatment that extend beyond just lifestyle changes [28].

Theme 4, screening and early diagnosis of obesity, and theme 5, treatment and management, addresses the importance of the continuum of care used in chronic diseases and aligns the language of obesity with the language and frameworks used in other policy prioritised NCDs [23, 24]. These themes focus on highlighting the importance of an obesity care cascade, where in the first step, populations are screened for obesity. When an individual is detected with signs of having obesity, the individual should be referred for an examination as to enable the early diagnosis of obesity. The early diagnosis of obesity refers to detecting an individual who is living with obesity as early as possible based on signs of this disease. Individuals who are diagnosed as living with obesity should get treatment where different actions are taken by individuals, families, communities, and healthcare professionals, to promote, maintain, control, and restore health in people living with obesity.

The obesity taxonomy underscores the imperative for a structured obesity vocabulary, given the lack of understanding about the scientific evidence of obesity as an adiposity-based chronic disease which is characterised by the function, total amount, and distribution of adipose tissue among various stakeholders involved in shaping and implementing health service delivery policies and practices and related regulatory implications. The confusion arises from the ambiguity surrounding whether obesity is viewed as an adiposity-based chronic disease and the prevalent use of terms like weight, weight management, overweight and BMI. This confusion permeates throughout research agendas, treatment protocols and policies, causing significant disruptions, particularly in health service delivery. As a result, policymakers are left with an insufficient understanding of what aspects they should legislate. The obesity taxonomy, derived through a rigorous three-round Delphi exercise, forms the basis for a common, precise, and scientifically accurate language related to obesity. The resulting taxonomy is relevant for educating as well as increasing awareness concerning obesity by delivering a tangible and searchable structured online lexicon that can be used by policymakers, health practitioners, patients, and health advocates. The taxonomy provides obesity terminology, that is consistent with other non-communicable diseases, is broken down into specific definitions, scope and context and is scientifically accurate. By establishing this clear framework, in a healthcare setting the taxonomy can facilitate clear communication among health care professionals and with health system users and may support collaboration and mutual understanding between health care professionals and policy makers. The use of appropriate language in the correct context is imperative in science and societal discussion in order to progress and deliver value for patients. Expert stakeholders consider that changes to language used at the point of care can support improved outcomes for both people living with obesity and for the functioning of the healthcare system itself [7].

This Delphi study had several strengths. Expert knowledge was gathered from a wide range of different stakeholders across different countries within the WHO Europe region. We reached experts from all relevant stakeholder groups across 30 different countries and sample size was more than four times the recommended lower limit threshold of 12 for a Delphi study. Given the extensive global reach of this field, the Delphi consensus technique, which can be executed online, was the fitting approach to gather these diverse viewpoints. Alongside identifying areas of consensus, the study highlighted areas where the field has some uncertainty, such as the theme of the definition of obesity, and the theme of causes, onset, and progression. These aspects might warrant further exploration and a broader interdisciplinary approach to effectively resolve these uncertainties. We used a panel of 14 experts specialising in obesity (the sounding board) to confirm the appropriateness of the Delphi methodology and to discuss the outcomes of the Delphi survey to verify the findings. Another notable strength of the study was the predetermined stringent definition of consensus, set a priori as agreement rates of 75% or higher for ratings 7–10, and agreement rates of less than 15% for ratings 0–3 [25] and no relevant comments based on a synthesis of free text responses. It is both a strength and a limitation that we accommodated free text responses. This invaluable input from the Delphi panellists shaped and improved the definitions, however, there remains the subjective nature of interpretation of the free text responses. Nevertheless, both core team members had to reach agreement in their interpretation, thus minimising the effects of subjectivity. While a strength of the study was its ability to access a network of colleagues in the field of obesity research, this may have introduced some response bias, as potentially only like-minded individuals participated in this study. However, the broad representation of stakeholders from different professional groups and countries counters this, but nonetheless we cannot preclude that the responses reflect a certain group of individuals. In this study, Delphi participants were invited to every round independent of response to previous rounds. However, an analysis of a previous Delphi study indicated that this invitation approach had no impact on the final results of this type of survey [29]. Another strength of our study is that, during rounds two and three, the group’s collective response to each statement and comments were presented to enable adequate reflection on group responses. This approach is considered optimal for achieving consensus [30]. Furthermore the statements were exclusively presented in English to the Delphi participants. We cannot preclude individuals less proficient in English may not have participated, thus resulting in less representation of views from non-English speaking populations. In addition, the distribution of stakeholders and countries may not have been evenly distributed across the three rounds of Delphi. There were lower rates of participation by individuals with the lived experience of obesity and individuals from Eastern European countries were observed across all three rounds of the Delphi process. Nonetheless, health care professionals and researchers were well-represented, as stakeholders from health literacy and other groups who can benefit from using the taxonomy were included.

## Conclusion

The obesity taxonomy, derived through a rigorous three-round Delphi exercise, forms the basis for a common, precise, and scientifically accurate language related to obesity. The taxonomy initiative harmonises the language used in other policy prioritised NCDs and is relevant for all stakeholder groups involved. The obesity taxonomy delivers a tangible and searchable structured online lexicon for policymakers, health care professionals, people living with obesity, researchers, and health system users to use as the basis of discussions for their work and interactions and will align and educate stakeholders on the language of chronic disease through the lens of obesity.

## Supplementary information

Supplemental material

## Data Availability

Information concerning data availability can be found in Appendix Table 4.
