# Supplementary material for: Providing a common language for obesity: the European Association for the Study of Obesity obesity taxonomy
Source: Int J Obes (Lond). 2024 Jun 20;49(2):182–91. doi: 10.1038/s41366-024-01565-9 (PMC11805702; doi:10.1038/s41366-024-01565-9)
Supplement: Supplementary file 1 — Supplemental material [file 41366_2024_1565_MOESM1_ESM.pdf]

Appendix table 1: Delphi round 1 results

EASO Obesity Taxonomy: round 1 Definitions	**Round 1**		
**Theme 1: Definition of obesity**	n	Rank 7-10	Suggestions
Concept: Obesity Definition			
• Definition: Obesity is defined as an abnormal or excessive fat accumulation that can impair health	62	93%	
• Scope: Obesity is an adiposity-based chronic disease which is characterized by the function, total amount and distribution of adipose tissue. Obesity is a disease that consists of different phenotypes.	63	89%	
• Context: The onset, development and progression of obesity can be influenced by a single or many causes or progressing factors.	64	95%	
Concept: Indicators of obesity			
• Definition: A metric describing the presence of the disease obesity at the population level.	62	79%	
• Scope: Indicators of obesity reflect the causes of the disease.	63	33%	*"I would consider indicators as describers of the actual situation without any links with the causes."*
• Context: The following non-exhaustive list of indicators of obesity can be examined: developmental and medical history, laboratory analyses, physical examination at the organ and system levels, mental health, medication intake, activities and tasks of daily living, human behaviours, participation in society, and human exposures.	61	88%	
Concept: Obesity disease staging frameworks			
• Definition: Obesity disease staging frameworks classify patients based on body composition and co-existing medical conditions, in order to determine the patients' health risk and optimise obesity treatment and management.	64	86%	*"This is not sufficient for me. The staging should also consider the physical capacity, disability, etc."*
• Scope: Staging frameworks are used in many diseases to help to determine disease severity and support clinical decision-making with regards to treatment and management.	64	94%	
• Context: Available obesity disease staging frameworks reflect disease frameworks used for other NCD's.	61	84%	
Concept: Pre-obesity			
• Definition: The state of health in which an individual shows pathological and metabolic signs that may be a precursor to obesity.	63	76%	*"If they have pathology or metabolic dysfunction they may already have obesity?"*
• Scope: The diagnosis of pre-obesity is reserved for individuals with altered adipose tissue and metabolic function.	64	59%	*"Alternatively is pre-obesity a state of excess or dysfunctional adipose tissue, but no evidence of metabolic or other health impairments?"*

• Context: The term overweight emphasizes weight and does not take into account the metabolic dysregulation of the disease. Therefore, overweight should not be used as a synonym for pre-obesity.	65	72%	*"Overweight is associated with metabolic dysregulation almost by definition"*
Concept: Signs<br>• Definition: Physical or mental parameters that may indicate the disease of obesity at the individual level<br>• Scope: Various metabolic and mental health impairments exist that may signal the presence of obesity in an individual.<br>• Context: At the individual level, an investigation of signs of obesity includes more than just measuring weight or BMI.	62<br>63<br>63	79%<br>79%<br>95%	*"and functional ??? a person is more than mind and body"*<br>*"I think mechanical or functional should be included with metabolic and mental health impairments"*

**Theme 2: Causes, Onset and Progression**	n	Rank 7-10	Suggestions
Concept: Causes<br>• Definition: An event, condition, characteristic or combination thereof which starts the onset of obesity.<br>• Scope: Causes of obesity are groups of events, conditions and characteristics that fundamentally alter the biology of the disease and go beyond lifestyle.<br>• Context: Causes of developing obesity may be biologically modifiable or biologically non-modifiable.	64<br>64<br>64	80%<br>82%<br>89%	*"I don't believe that "causes" alter "the disease", they produce it."*
Concept: Onset<br>• Definition: The disease onset is a change in usual health status with signs directly attributable to obesity.<br>• Scope: The onset of obesity is the start of the processes that lead to the biological manifestation of obesity.<br>• Context: The biological processes that provoke the onset of obesity may be ongoing for a long period of time before they are detected.	64<br>62<br>64	75%<br>77%<br>94%	*"I don't think this would be a helpful way to look at it as it could miss many cases where individuals are looking for changes which don't always happen until later disease stages."*<br>*"I would argue that the onset of obesity is the biological manifestation of obesity.*<br>*manifestations are not only biological could be mechanical, psychological."*
Concept: Progression factors<br>• Definition: An event, condition, or characteristic or combination thereof that exacerbates obesity.<br>• Scope: Groups of events, conditions and characteristics that exacerbate the progression of obesity by fundamentally altering the biology of the disease and go beyond general lifestyle considerations.	64<br>65<br>62	77%<br>85%<br>85%	*"This definition is talking about contributors, but not about "progression" itself. "Progression" is more the "exacerbation" by any event, condition or characteristic."*<br>*"Very complicated definition. Difficult to understand"*

• Context: Factors that cause the progression of obesity may be biologically modifiable or biologically non-modifiable.			

EASO Obesity Taxonomy: round 1 Definitions	**Round 1**		
**Theme 3: Obesity prevention**	n	Rank 7-10	Suggestions
Concept: Health promotion<br>• Definition: Health promotion is the process of enabling and supporting people and populations to maximise their health and quality of life.<br>• Scope: Health promotion is generally a behavioral approach to supporting a healthy lifestyle for all.<br>• Context: Health promotion is delivered to the general public and not only those who might be at risk of obesity.	65<br>63<br>64	97%<br>80%<br>90%	
Concept: Primary prevention<br>• Definition: Primary prevention aims to prevent the disease of obesity before it ever occurs.<br>• Scope: Primary prevention targets risk factors in the general population or at the individual level compared to health promotion which applies to the whole population.<br>• Context: Primary prevention is distinct from secondary prevention, which means early detection, diagnosis and treatment as to stop the progression of obesity and the development of health consequences, and tertiary prevention which means treating and managing the disease of obesity to reduce its long lasting effects.	64<br>63<br>63	92%<br>80%<br>92%	 *"The statement seems to make a comparison/describe a contrast, but then we have "general population or ..." vs. "whole population"."*

EASO Obesity Taxonomy: round 1 Definitions	**Round 1**		
**Theme 4: Screening and Early diagnosis**	n	Rank 7-10	Suggestions
Concept: Obesity Screening<br>• Definition: Screening for obesity refers to the investigation of obesity indicators in populations as to identify individuals with signs of having obesity.<br>• Scope: Elements to consider include a person's age, biological sex, and ethnic background.<br>• Context: Obesity screening can lead to the identification of factors that change the likelihood of developing obesity and use of this knowledge to prevent or lessen obesity by modifying these factors.	62<br>61<br>63	87%<br>82%<br>81%	 *"Education? Socioeconomic background?"*
Concept: Obesity early diagnosis<br>• Definition: Early diagnosis of obesity refers to detecting an individual who is living with obesity as early as possible based on signs of this disease.	62<br>57	90%<br>79%	 *"It should be made clear what is meant with "consider" in this case."*

• Scope: Elements to consider include a person's age, biological sex, and ethnic background.<br><br>• Context: Early diagnosis of obesity can lead to better control of disease and to better patient-centered health outcomes, medical outcomes, and socio-economic outcomes in the long term.	61	93%	

EASO Obesity Taxonomy: round 1 Definitions	**Round 1**		
**Theme 5: Treatment and management**	n	Rank 7-10	Suggestions
Concept: Obesity treatment<br>• Definition: Medical care given to a patient living with obesity.	65	78%	*"It is not just medical care. A more appropriate definition would be healthcare given to a patient living with obesity."*
• Scope: Treatment options for obesity or a combination thereof include: Therapeutic physical activity and rehabilitation, Therapeutic nutrition, Psychological therapy, Pharmacotherapy, Metabolic and bariatric surgery	65	94%	
• Context: Medical obesity treatment options take a multidisciplinary and holistic approach, and if possible, are person-centred and individualised. Obesity treatment options can be categorized as acute or long-term and consider the fluctuating nature of the disease.	63	92%	
Concept: Obesity management<br>• Actions taken by individuals, families and communities to promote, maintain and restore health in people living with obesity.	64	88%	
• Scope: Obesity management consists of different levels including: supported self-management, clinical support, informal support and support from the overarching health and social security system.	65	95%	
• Context: Obesity management focusses on rebalancing the biological dysregulation, improving signs and symptoms and thus optimising patient-centred health outcomes, medical outcomes and socio-economic outcomes. Weight management may be a component of this.	63	87%	
Concept: Obesity treatment and management outcomes<br>• Evaluation undertaken to assess the results or consequences of treating and managing obesity.	63	89%	
• Scope: When treating or managing obesity, patient-centred health outcomes, medical outcomes, and socio-economic outcomes are assessed.	63	94%	
• Context: Obesity treatment and management outcomes go beyond weight.	64	98%	

Concept: Shared-decision making<br>• Definition: A process in which both the patient and the healthcare professional work together to decide the best plan of obesity care for the patient.	62	98%	
• Scope: The conversation brings together: the clinician's expertise, such as treatment options, evidence, risks and benefits; what the patient knows best, their preferences, personal circumstances, goals, values and beliefs.	62	98%	
• Context: Shared decision-making forms the basis of a clinical consultation. Implementation of this process is useful for complex medical decisions.	63	98%	

EASO Obesity Taxonomy: round 1 Definitions	**Round 1**		
**Theme 6: Obesity consequences**	n	Rank 7-10	Suggestions
Concept: Obesity health complications<br>• Definition: Obesity is a gateway disease to a range of medical and mental complications.	62	92%	
• Scope: 200 + complications are associated with obesity including: Type 2 diabetes, heart disease and cancer.	61	94%	
• Context: Obesity was regarded as a comorbidity of many non-communicable diseases whereas now these diseases are seen as medical complications of obesity.	63	89%	
Concept: Obesity socio-economic consequences<br>• Definition: Obesity can harm an individual's education, income, job opportunities and value creation.	62	98%	
• Scope: Obesity impacts individuals at several socio-economic levels and decreases their quality adjusted life years.	57	97%	
• Context: Obesity places a significant pressure on public and specialised health institutions in terms of costs and quality of services. Which may lead to a deficiency in the services provided to society members in general.	61	83%	*"This is probably factually correct but I am unsure of the tone. A reader of this statement may perceive this as saying that people with obesity are depriving other people (without obesity) of access to healthcare services"*

Appendix table 2: Delphi round 2 results

EASO Obesity Taxonomy: round 2 Definitions	**Round 2**		
**Theme 1: Definition of obesity**	n	Rank 7-10	Suggestions
Concept: Indicators of obesity<br>• Scope: Indicators of obesity signal the presence of the disease at the population level	49	84%	
Concept: Obesity disease staging frameworks	48	83%	

• Definition: Obesity disease staging frameworks classify people based on obesity signs and indicators, to determine an individual's health risk and optimise obesity treatment and management.			
Concept: Pre-obesity<br>• Definition: The state of health in which an individual exhibits changes in the function, total amount and/or distribution of adipose tissue that may be a precursor to obesity.<br><br>• Scope: The state of health in individuals with altered function, total amount and/or distribution of adipose tissue before pathological signs emerge.<br><br>• Context: Overweight is not a synonym for pre-obesity as it does not reflect the dysregulation of adipose tissue.	49<br><br>49<br><br>49	82%<br><br>76%<br><br>71%	*"I agree that overweight is not the same as pre-obesity, but I believe in some cases (not all!!!) it does 'reflect' the dysregulation of adipose tissue"*
Concept: Signs<br>• Definition: A metric describing the presence of the disease obesity at the individual level.<br><br>• Scope: Physiological, functional or psychological health impairments exist that may signal the presence of obesity in an individual.	49<br><br>48	74%<br><br>85%	*"Using the term 'A metric' is vague. A sign is basically a manifestation of disease that can be perceived by a health care professional (it can be physical, mental or a lab test)."*

EASO Obesity Taxonomy: round 2 Definitions	**Round 2**		
**Theme 2: Causes, Onset and Progression**	n	Rank 7-10	Suggestions
Concept: Causes<br>• Scope: Factors that produce malfunctioning adipose tissue, an abnormal distribution and/ or an excessive amount of adipose tissue.	52	81%	
Concept: Onset<br>• Definition: The disease onset is a given moment in time when changes occur that alter the function, distribution and/or amount of adipose tissue.<br><br>• Scope: The onset of obesity is the start of the processes that lead to the manifestation of obesity.	51<br><br>52	75%<br><br>79%	
Concept: Progression factors:<br>• Scope: Factors that change the severity of obesity by altering the biology of the disease.	52	73%	*"much better, but consider to change it to "increase the severity "instead of "change the severity "because a change towards a lower severity is not disease progression"*

EASO Obesity Taxonomy: round 2 Definitions	**Round 2**		
**Theme 3: Obesity prevention**	n	Rank 7-10	Suggestions
Concept: Primary prevention			

• Scope: Primary prevention targets risk factors of obesity compared to health promotion which enables people to increase control over, and to improve, their health.	50	84%	

EASO Obesity Taxonomy: round 2 Definitions	**Round 2**		
**Theme 4: Screening and Early diagnosis**	n	Rank 7-10	Suggestions
<u>Concept: Obesity Screening</u><br>• Scope: Elements to consider when screening for indicators for obesity may include a person's age, biological sex, body composition, ethnic background, family history, pre-existing medical-conditions and among others.	50	80%	
<u>Concept: Obesity early diagnosis</u><br>• Scope: Elements to consider when evaluating signs for the early diagnosis of obesity may include a person's age, biological sex, body composition, ethnic background, family history, pre-existing medical-conditions and among others.	50	84%	

EASO Obesity Taxonomy: round 2 Definitions	**Round 2**		
**Theme 5: Treatment and management**	n	Rank 7-10	Suggestions
<u>Concept: Obesity treatment</u><br>• Definition: Healthcare given to a patient living with obesity by a healthcare professional.	53	94%	

EASO Obesity Taxonomy: round 2 Definitions	**Round 2**		
**Theme 6: Obesity consequences**	n	Rank 7-10	Suggestions
<u>Concept: Obesity socio-economic consequences</u><br>• Context: Obesity places a significant pressure on public and specialised health institutions in terms of costs and quality of services.	50	88%	

<u>Appendix table 3: Delphi round 3 results</u>

EASO Obesity Taxonomy: round 3 Definitions	**Round 3**		
**Theme 1: Definition of obesity**	n	Rank 7-10	Suggestions
<u>Concept: Pre-obesity</u><br>• Context: Overweight does not always reflect the dysregulation of adipose tissue in individuals and therefore is not a synonym for pre-obesity.	52	81%	
<u>Concept: Signs</u><br>• Definition: A manifestation of the disease obesity at the individual level.	52	87%	

EASO Obesity Taxonomy: round 3 Definitions	**Round 3**		
**Theme 2: Causes, Onset and Progression**	n	Rank 7-10	Suggestions
<u>Concept: Progression factors:</u><br>• Scope: Factors that increase the severity of obesity by altering the biology of the disease.	51	92%	

Appendix table 4: Data sharing

Data sharing statements	Answer
Will individual participant data be available?	Yes
What data in particular will be shared?	All of the individual participant data collected during the study, after de-identification
What other documents will be available?	Study protocol
When will data be available (start and end dates)?	Immediately following publication; no end date
With whom?	Anyone who wishes to access the data
For what types of analyses?	Any purpose
By what mechanism will data be made available?	Data will be made available through email.

Appendix 5: Delphi survey

EASO
European Association for the Study of Obesity

EASO Obesity Taxonomy

Informed consent

Study title: European Association for the Study of Obesity (EASO) Obesity Taxonomy

The EASO Policy group is inviting you to participate in an adapted Delphi exercise to formulate a taxonomy, or a classification scheme, for obesity.

What is the purpose of this study?
The purpose of this survey is to provide the basics for a working vocabulary about obesity in terms of definition, scope and contextual usage for key concepts and related terminology within the context of a non-communicable disease (NCD) policy framework.

What is expected from you?
The adapted Delphi exercise will consist of at least one survey and potentially participation in a meeting. In the survey, you will be asked to rank different concepts in terms of their definition, scope of what they cover, and context about how they are used. These concepts will be ranked based on how much you agree with the statement using a visual analogue scale (from 0 'completely do not agree' to 10 'completely agree'). An optional free-text box is included with each statement in which you elaborate upon your ranking or add suggestions. After the answers are analyzed, you may be invited to participate in the negotiation meeting and/or to fill in additional rounds of the survey. Participation is entirely voluntary.

Estimated number of participants:
In total, more than 100 participants across different stakeholder sectors, representing healthcare professionals, policymakers, researchers, industry, payor community and patient advocates will be invited to fill in the survey.

How long will it take?
Completing the survey will take approximately 45 minutes of your time.

Maintenance of privacy
We ask that you submit your name, which stakeholder group you represent and your country. We ask for your name so that we can invite you to participate in the negotiation meeting. All answers will be separated from your personal information. The resulting data set will be anonymized, and results will only be presented at the aggregate level. All data will be stored in an electronic database on a password-

protected encrypted computer.

Funding:
This project has been funded by the European Association for the Study of Obesity, and we acknowledge additional financial support from Eli Lilly and Company.

Should you have any questions, please do not hesitate to contact Jacqueline Bowman-Busato (jbowman@easo.org) or Jennifer L. Baker (jennifer.lyn.baker@regionh.dk)

Agreement to Participate:
Your participation is completely voluntary and you can withdraw at any time.

If you agree with these terms, please fill in your name (optional, but without it we cannot invite you to the negotiation meeting), which stakeholder group you represent and your country and click the button below to start.

1. Please fill in your name and email address

[ ]

*** 2. Please fill in your stakeholder group**

- [ ] Clinician
- [ ] Nurse
- [ ] Dietitian
- [ ] Physiotherapist
- [ ] Patient advocate
- [ ] Researcher
- [ ] Policymaker Payor
- community Industry
- [ ]
- [ ]
- [ ] Other (please specify)

[ ]

*** 3. Please fill in your country**

[ ]

EASO
European Association for the Study of Obesity

EASO Obesity Taxonomy

Pillar 1: Definition of obesity

Pillar 1 consists of 5 different concepts. For each concept, please rank definition, scope and context on level of agreement by using the visual analogue scale (from 0 'completely not agree' to 10 'completely agree'). A free-text box was added within each statement, providing the opportunity to elaborate or explain responses.

Concept 1: Obesity definition

1. Definition: Obesity is defined as an abnormal or excessive fat accumulation that can impair health

0	1	2	3	4	5	6	7	8	9	10
○	○	○	○	○	○	○	○	○	○	○

Remarks?

2. Scope: Obesity is an adiposity-based chronic disease which is characterized by the function, total amount and distribution of adipose tissue. Obesity is a disease that consists of different phenotypes.

0	1	2	3	4	5	6	7	8	9	10
○	○	○	○	○	○	○	○	○	○	○

Remarks?

3. **Context: The onset, development and progression of obesity can be influenced by a single or many causes or progressing factors.**

0	1	2	3	4	5	6	7	8	9	10
○	○	○	○	○	○	○	○	○	○	○

Remarks?

Concept 2: Indicators of obesity

4. **Definition: A metric describing the presence of the disease obesity at the population level.**

0	1	2	3	4	5	6	7	8	9	10
○	○	○	○	○	○	○	○	○	○	○

Remarks?

5. **Scope: Indicators of obesity reflect the causes of the disease.**

0	1	2	3	4	5	6	7	8	9	10
○	○	○	○	○	○	○	○	○	○	○

Remarks?

6. **Context: The following non-exhaustive list of indicators of obesity can be examined: developmental and medical history, laboratory analyses, physical examination at the organ and system levels, mental health, medication intake, activities and tasks of daily living, human behaviours, participation in society, and human exposures.**

0	1	2	3	4	5	6	7	8	9	10
○	○	○	○	○	○	○	○	○	○	○

Remarks?

Concept 3: Obesity disease staging frameworks

7. **Definition: Obesity disease staging frameworks classify patients based on body composition and co-existing medical conditions, in order to determine the patients' health risk and optimise obesity treatment and management.**

0	1	2	3	4	5	6	7	8	9	10
○	○	○	○	○	○	○	○	○	○	○

Remarks?

8. **Scope: Staging frameworks are used in many diseases to help to determine disease severity and support clinical decision-making with regards to treatment and management.**

0	1	2	3	4	5	6	7	8	9	10
○	○	○	○	○	○	○	○	○	○	○

Remarks?

9. **Context: Available obesity disease staging frameworks reflect disease frameworks used for other NCD's.**

0	1	2	3	4	5	6	7	8	9	10
○	○	○	○	○	○	○	○	○	○	○

Remarks?

Concept 4: Pre-obesity

10. **Definition: The state of health in which an individual shows pathological and metabolic signs that may be a precursor to obesity.**

0	1	2	3	4	5	6	7	8	9	10
○	○	○	○	○	○	○	○	○	○	○

Remarks?

11. **Scope: The diagnosis of pre-obesity is reserved for individuals with altered adipose tissue and metabolic function.**

0	1	2	3	4	5	6	7	8	9	10
○	○	○	○	○	○	○	○	○	○	○

Remarks?

12. **Context: The term overweight emphasizes weight and does not take into account the metabolic dysregulation of the disease. Therefore, overweight should not be used as a synonym for pre-obesity.**

0	1	2	3	4	5	6	7	8	9	10
○	○	○	○	○	○	○	○	○	○	○

Remarks?

Concept 5: Signs

13. **Definition: Physical or mental parameters that may indicate the disease of obesity at the individual level.**

0	1	2	3	4	5	6	7	8	9	10
○	○	○	○	○	○	○	○	○	○	○

Remarks?

14. **Scope: Various metabolic and mental health impairments exist that may signal the presence of obesity in an individual.**

0	1	2	3	4	5	6	7	8	9	10
○	○	○	○	○	○	○	○	○	○	○

Remarks?

15. Context: At the individual level, an investigation of signs of obesity includes more than just measuring weight or BMI.

0	1	2	3	4	5	6	7	8	9	10
○	○	○	○	○	○	○	○	○	○	○

Remarks?

EASO
European Association for the Study of Obesity

EASO Obesity Taxonomy

Pillar 2: Causes, onset and progression

Pillar 2 consists of 3 different concepts. For each concept, please rank definition, scope and context on level of agreement by using the visual analogue scale (from 0 'completely not agree' to 10 'completely agree'). A free-text box was added within each statement, providing the opportunity to elaborate or explain responses.

Concept 1: Causes

1. Definition: An event, condition, characteristic or combination thereof which starts the onset of obesity.

0	1	2	3	4	5	6	7	8	9	10
○	○	○	○	○	○	○	○	○	○	○

Remarks?

2. Scope: Causes of obesity are groups of events, conditions and characteristics that fundamentally alter the biology of the disease and go beyond lifestyle.

0	1	2	3	4	5	6	7	8	9	10
○	○	○	○	○	○	○	○	○	○	○

Remarks?

3. Context: Causes of developing obesity may be biologically modifiable or biologically non- modifiable.

0	1	2	3	4	5	6	7	8	9	10
○	○	○	○	○	○	○	○	○	○	○

Remarks?

Concept 2: Onset

4. Definition: The disease onset is a change in usual health status with signs directly attributable to obesity.

0	1	2	3	4	5	6	7	8	9	10
○	○	○	○	○	○	○	○	○	○	○

Remarks?

5. Scope: The onset of obesity is the start of the processes that lead to the biological manifestation of obesity.

0	1	2	3	4	5	6	7	8	9	10
○	○	○	○	○	○	○	○	○	○	○

Remarks?

6. Context: The biological processes that provoke the onset of obesity may be ongoing for a long period of time before they are detected.

0	1	2	3	4	5	6	7	8	9	10
○	○	○	○	○	○	○	○	○	○	○

Remarks?

Concept 3: Progression

7. Definition: An event, condition, or characteristic or combination thereof that exacerbates obesity.

0	1	2	3	4	5	6	7	8	9	10
○	○	○	○	○	○	○	○	○	○	○

Remarks?

8. **Scope: Groups of events, conditions and characteristics that exacerbate the progression of obesity by fundamentally altering the biology of the disease and go beyond general lifestyle considerations.**

0	1	2	3	4	5	6	7	8	9	10
○	○	○	○	○	○	○	○	○	○	○

Remarks?

9. **Context: Factors that cause the progression of obesity may be biologically modifiable or biologically non-modifiable.**

0	1	2	3	4	5	6	7	8	9	10
○	○	○	○	○	○	○	○	○	○	○

Remarks?

EASO
European Association for the Study of Obesity

EASO Obesity Taxonomy

Pillar 3: Obesity prevention

Pillar 3 consists of 2 different concepts. For each concept, please rank definition, scope and context on level of agreement by using the visual analogue scale (from 0 'completely not agree' to 10 'completely agree'). A free-text box was added within each statement, providing the opportunity to elaborate or explain responses.

Concept 1: Health promotion

1. Definition: Health promotion is the process of enabling and supporting people and populations to maximise their health and quality of life.

0	1	2	3	4	5	6	7	8	9	10
○	○	○	○	○	○	○	○	○	○	○

Remarks?

2. Scope: Health promotion is generally a behavioral approach to supporting a healthy lifestyle for all.

0	1	2	3	4	5	6	7	8	9	10
○	○	○	○	○	○	○	○	○	○	○

Remarks?

3. Context: Health promotion is delivered to the general public and not only those who might be at risk of obesity.

0	1	2	3	4	5	6	7	8	9	10
○	○	○	○	○	○	○	○	○	○	○

Remarks?

Concept 2: Primary prevention

4. Definition: Primary prevention aims to prevent the disease of obesity before it ever occurs.

0	1	2	3	4	5	6	7	8	9	10
○	○	○	○	○	○	○	○	○	○	○

Remarks?

5. Scope: Primary prevention targets risk factors in the general population or at the individual level compared to health promotion which applies to the whole population.

0	1	2	3	4	5	6	7	8	9	10
○	○	○	○	○	○	○	○	○	○	○

Remarks?

6. Context: Primary prevention is distinct from secondary prevention, which means early detection, diagnosis and treatment as to stop the progression of obesity and the development of health consequences, and tertiary prevention which means treating and managing the disease of obesity to reduce its long lasting effects.

0	1	2	3	4	5	6	7	8	9	10
○	○	○	○	○	○	○	○	○	○	○

Remarks?

EASO
European Association for the Study of Obesity

EASO Obesity Taxonomy

Pillar 4: Screening and early diagnosis

Pillar 4 consists of 2 different concepts. For each concept, please rank definition, scope and context on level of agreement by using the visual analogue scale (from 0 'completely not agree' to 10 'completely agree'). A free-text box was added within each statement, providing the opportunity to elaborate or explain responses.

Concept 1: Screening

1. Definition: Screening for obesity refers to the investigation of obesity indicators in populations as to identify individuals with signs of having obesity.

0	1	2	3	4	5	6	7	8	9	10
○	○	○	○	○	○	○	○	○	○	○

Remarks?

2. Scope: Elements to consider include a person's age, biological sex, and ethnic background.

0	1	2	3	4	5	6	7	8	9	10
○	○	○	○	○	○	○	○	○	○	○

Remarks?

3. **Context: Obesity screening can lead to the identification of factors that change the likelihood of developing obesity and use of this knowledge to prevent or lessen obesity by modifying these factors.**

0	1	2	3	4	5	6	7	8	9	10
○	○	○	○	○	○	○	○	○	○	○

Remarks?

Concept 2: Early diagnosis

4. **Definition: Early diagnosis of obesity refers to detecting an individual who is living with obesity as early as possible based on signs of this disease.**

0	1	2	3	4	5	6	7	8	9	10
○	○	○	○	○	○	○	○	○	○	○

Remarks?

5. **Scope: Elements to consider include a person's age, biological sex, and ethnic background.**

0	1	2	3	4	5	6	7	8	9	10
○	○	○	○	○	○	○	○	○	○	○

Remarks?

6. **Context: Early diagnosis of obesity can lead to better control of disease and to better patient-centered health outcomes, medical outcomes, and socio-economic outcomes in the long term.**

0	1	2	3	4	5	6	7	8	9	10
○	○	○	○	○	○	○	○	○	○	○

Remarks?

EASO
European Association for the Study of Obesity

EASO Obesity Taxonomy

Pillar 5: Treatment and management

Pillar 5 consists of 4 different concepts. For each concept, please rank definition, scope and context on level of agreement by using the visual analogue scale (from 0 'completely not agree' to 10 'completely agree'). A free-text box was added within each statement, providing the opportunity to elaborate or explain responses.

Concept 1: Obesity treatment

1. Definition: Medical care given to a patient living with obesity.

0	1	2	3	4	5	6	7	8	9	10
○	○	○	○	○	○	○	○	○	○	○

Remarks?

2. Scope: Treatment options for obesity or a combination thereof include:

- **Therapeutic physical activity and rehabilitation**
- **Therapeutic nutrition**
- **Psychological therapy**
- **Pharmacotherapy**
- **Metabolic and bariatric surgery**

0	1	2	3	4	5	6	7	8	9	10
○	○	○	○	○	○	○	○	○	○	○

Remarks?

3. **Context: Medical obesity treatment options take a multidisciplinary and holistic approach, and if possible, are person-centred and individualised. Obesity treatment options can be categorized as acute or long-term and consider the fluctuating nature of the disease.**

0	1	2	3	4	5	6	7	8	9	10
○	○	○	○	○	○	○	○	○	○	○

Remarks?

Concept 2: Obesity management

4. **Definition: Actions taken by individuals, families and communities to promote, maintain and restore health in people living with obesity.**

0	1	2	3	4	5	6	7	8	9	10
○	○	○	○	○	○	○	○	○	○	○

Remarks?

5. **Scope: Obesity management consists of different levels including: supported self- management, clinical support, informal support and support from the overarching health and social security system.**

0	1	2	3	4	5	6	7	8	9	10
○	○	○	○	○	○	○	○	○	○	○

Remarks?

6. **Context: Obesity management focusses on rebalancing the biological dysregulation, improving signs and symptoms and thus optimising patient-centred health outcomes, medical outcomes and socio-economic outcomes. Weight management may be a component of this.**

0	1	2	3	4	5	6	7	8	9	10
○	○	○	○	○	○	○	○	○	○	○

Remarks?

Concept 3: Obesity treatment and management outcomes

7. **Definition: Evaluation undertaken to assess the results or consequences of treating and managing obesity.**

0	1	2	3	4	5	6	7	8	9	10
○	○	○	○	○	○	○	○	○	○	○

Remarks?

8. **Scope: When treating or managing obesity, patient-centered health outcomes, medical outcomes, and socio-economic outcomes are assessed.**

0	1	2	3	4	5	6	7	8	9	10
○	○	○	○	○	○	○	○	○	○	○

Remarks?

9. **Context: Obesity treatment and management outcomes go beyond weight.**

0	1	2	3	4	5	6	7	8	9	10
○	○	○	○	○	○	○	○	○	○	○

Remarks?

Concept 4: Shared decision-making

10. **Definition: A process in which both the patient and the healthcare professional work together to decide the best plan of obesity care for the patient.**

0	1	2	3	4	5	6	7	8	9	10
○	○	○	○	○	○	○	○	○	○	○

Remarks?

11. **Scope: The conversation brings together:**

- **the clinician's expertise, such as treatment options, evidence, risks and benefits**
- **what the patient knows best: their preferences, personal circumstances, goals, values and beliefs**

0	1	2	3	4	5	6	7	8	9	10
○	○	○	○	○	○	○	○	○	○	○

Remarks?

12. **Context: Shared decision-making forms the basis of a clinical consultation. Implementation of this process is useful for complex medical decisions.**

0	1	2	3	4	5	6	7	8	9	10
○	○	○	○	○	○	○	○	○	○	○

Remarks?

EASO
European Association for the Study of Obesity

EASO Obesity Taxonomy

Pillar 6: Obesity consequences

Pillar 6 consists of 2 different concepts. For each concept, please rank definition, scope and context on level of agreement by using the visual analogue scale (from 0 'completely not agree' to 10 'completely agree'). A free-text box was added within each statement, providing the opportunity to elaborate or explain responses.

Concept 1: Obesity health complications

1. Definition: Obesity is a gateway disease to a range of medical and mental complications.

0	1	2	3	4	5	6	7	8	9	10
○	○	○	○	○	○	○	○	○	○	○

Remarks?

2. Scope: 200 + complications are associated with obesity including: Type 2 diabetes, heart disease and cancer.

0	1	2	3	4	5	6	7	8	9	10
○	○	○	○	○	○	○	○	○	○	○

Remarks?

3. Context: Obesity was regarded as a comorbidity of many non-communicable diseases whereas now these diseases are seen as medical complications of obesity.

0	1	2	3	4	5	6	7	8	9	10
○	○	○	○	○	○	○	○	○	○	○

Remarks?

Concept 1: socio-economic consequences

4. Definition: Obesity can harm an individual's education, income, job opportunities and value creation.

0	1	2	3	4	5	6	7	8	9	10
○	○	○	○	○	○	○	○	○	○	○

Remarks?

5. Scope: Obesity impacts individuals at several socio-economic levels and decreases their quality adjusted life years.

0	1	2	3	4	5	6	7	8	9	10
○	○	○	○	○	○	○	○	○	○	○

Remarks?

6. Context: Obesity places a significant pressure on public and specialised health institutions in terms of costs and quality of services. Which may lead to a deficiency in the services provided to society members in general.

0	1	2	3	4	5	6	7	8	9	10
○	○	○	○	○	○	○	○	○	○	○

Remarks?

EASO Obesity Taxonomy

Thank you for completing the suvery!

The results will be presented at our second engagement meeting on 24 March.
